# A study protocol for the N-ICE trial: A randomised double-blind placebo-controlled study of the safety and efficacy of *N*-acetyl-cysteine (NAC) as a pharmacotherapy for methamphetamine (“ice”) dependence

**DOI:** 10.1186/s13063-019-3450-0

**Published:** 2019-06-04

**Authors:** Rebecca McKetin, Olivia M. Dean, Alyna Turner, Peter J. Kelly, Brendan Quinn, Dan I. Lubman, Paul Dietze, Gregory Carter, Peter Higgs, Amanda L. Baker, Barbara Sinclair, David Reid, Victoria Manning, Nina te Pas, Wenbin Liang, Tamsin Thomas, Ramez Bathish, Margaret Kent, Dayle Raftery, Shalini Arunogiri, Frank Cordaro, Harry Hill, Michael Berk

**Affiliations:** 1 National Drug Research institute, Curtin University, GPO Box 1987, Perth, WA 6845 Australia; 2 IMPACT Strategic Research Centre, School of Medicine, Deakin University, Barwon Health, Geelong, Australia; 3 Florey Institute for Neuroscience and Mental Health, University of Melbourne, Melbourne, Australia; 4 Department of Psychiatry, University of Melbourne, Parkville, Australia; 5 School of Medicine and Public Health, University of Newcastle, Callaghan, Australia; 6 Illawarra Health and Medical Research Institute and School of Psychology, University of Wollongong, Wollongong, Australia; 7 Behaviours and Health Risks Program, Burnet Institute, Melbourne, Australia; 8 Eastern Health Clinical School, Faculty of Medicine, Nursing & Health Sciences, Monash University, Melbourne, Australia; 9 Turning Point, Eastern Health, Richmond, Australia; 10 School of Psychology and Public Health, La Trobe University, Melbourne, Australia; 11 Drug and Alcohol Services, Illawarra Shoalhaven Local Health District, Wollongong, Australia; 12 Barwon Health Drug and Alcohol Services, Geelong, Australia; 13 Orygen, The National Centre of Excellence in Youth Mental Health, University of Melbourne, Parkville, Australia

**Keywords:** substance use disorders, methamphetamine, *N*-acetylcysteine, clinical trial, craving, withdrawal, psychosis, aggression, depression, suicide

## Abstract

**Background:**

There are currently no approved pharmacotherapies for managing methamphetamine dependence. *N*-acetylcysteine (NAC) has been found to reduce the craving for methamphetamine and other drugs, but its effect on methamphetamine use and other clinically related endpoints are uncertain. The N-ICE trial is evaluating the safety and efficacy of NAC as a take-home pharmacotherapy for methamphetamine dependence.

**Methods/design:**

This is a two-arm parallel double-blind placebo-controlled three-site randomised trial (ratio 1:1) using permuted block randomisation, with variable block sizes. It is stratified by site, sex and whether the methamphetamine is injected or not. Participants (*N* = 180; 60 per site) need to be dependent on methamphetamine, interested in reducing their methamphetamine use and not currently receiving treatment for substance use disorders. The trial is being conducted in outpatient settings in Melbourne, Geelong and Wollongong, Australia. Participants will receive either 2400 mg oral NAC or a matched placebo, delivered as a take-home medication for 12 weeks. Two 600 mg capsules are self-administered in the morning and two more in the evening. Adherence is being monitored using eCAP™ medication bottle lids, which record the date and time of each occasion the bottle is opened. The primary outcome is methamphetamine use during the 12-week trial medication period, measured as (a) days of use, assessed using the timeline followback, and (b) methamphetamine-positive saliva tests, taken weekly. Secondary measures include weekly assessment of methamphetamine craving, severity of methamphetamine dependence, methamphetamine withdrawal symptoms and psychiatric symptoms (depression, suicidality, psychotic symptoms and hostility). Adverse events are monitored at each weekly assessment. Tolerability is assessed using the Treatment Satisfaction Questionnaire for Medication.

**Discussion:**

The N-ICE trial is the first clinical trial to assess whether NAC can reduce methamphetamine use. This trial will improve our understanding of the potential utility of NAC in managing methamphetamine dependence and clinically related outcomes. If found to be effective, take-home NAC could be a potentially scalable and affordable pharmacotherapy option for treating methamphetamine dependence.

**Trial registration:**

Australian and New Zealand Clinical Trials Registry, ACTRN12618000366257. Registered on 29 May 2018.

**Electronic supplementary material:**

The online version of this article (10.1186/s13063-019-3450-0) contains supplementary material, which is available to authorized users.

## Introduction

Methamphetamine (“ice”) use has become a global health concern [1] and methamphetamine dependence is now a major contributor to the burden of disease [2]. Hence, there is an urgent need for scalable and cost-effective treatment options [3]. However, currently there are no approved pharmacotherapies for treating methamphetamine dependence or withdrawal [3, 4], or its psychiatric sequelae [5, 6], with existing treatment options entirely reliant on psychosocial interventions [3]. Although these can provide a modest benefit [7, 8], they can be resource intensive to deliver [9] and are typically accessed by only a minority of consumers, particularly in rural and resource-poor settings [10, 11]. Pharmacotherapy may be a potentially scalable and cost-effective treatment option that could dramatically increase treatment coverage.

*N*-acetylcysteine (NAC) is a novel non-agonist pharmacotherapy that targets changes in glutamate function in the nucleus accumbens, which are thought to underpin drug craving and drug seeking [12]. NAC has been shown to reduce cravings for various drugs, including cocaine, tobacco, cannabis and methamphetamine [13]. However, evidence of reduced substance use is mixed [14, 15] and derives mainly from preclinical research and open-label and small-scale trials [16–18]. One small-scale crossover trial has demonstrated significant reductions in the craving for methamphetamine [19], but no trials have explored whether such reductions in cravings translate into clinically meaningful reductions in methamphetamine use amongst people who are dependent on the drug.

NAC may also convey a therapeutic benefit in managing the psychiatric sequelae associated with methamphetamine dependence. In addition to its potential for reducing drug cravings, pre-exposure to NAC reduces methamphetamine-related neurotoxicity [20]. These neurotoxic changes manifest as reduced dopamine and serotonin transporter density in the frontal, striatal and limbic regions [21, 22], which correlate with psychiatric symptoms, including hostility, in chronic methamphetamine users [23, 24]. Previous clinical trials have suggested that NAC can reduce psychiatric symptoms in the context of bipolar disorder, schizophrenia and major depression [25]. However, the potential therapeutic mitigation of psychiatric symptoms amongst people who use methamphetamine [26, 27] has not been explored.

NAC’s well-established safety profile means that it can be delivered as a take-home medication [28], making it a potentially scalable and cost-effective treatment option. It is no longer under any patent restrictions, further enhancing its roll-out and take-up. Known adverse reactions include gastrointestinal upsets and rash [29], which have not been significantly elevated relative to the placebo in current NAC trials for substance use disorders [30]. The seemingly generic effect of NAC on drug craving suggests that it may also be helpful in managing polysubstance dependence.

### Aims and hypotheses

The aim of the current trial is to test the safety and efficacy of NAC as a pharmacotherapy for methamphetamine dependence using a double-blindplacebo-controlled randomised controlled trial. The trial will recruit 180 participants, who will receive either 12 weeks of oral NAC (2400 mg daily) or matched placebo. This is a phase IIb trial that is powered to confirm whether NAC has a clinically relevant benefit on methamphetamine use. The comparator condition is a matched placebo, with all participants provided with a minimal form of intervention (a self-help booklet), due to the lack of readily available standardised effective treatment options for methamphetamine use.

The primary hypothesis is that daily oral NAC delivered as a take-home medication will reduce methamphetamine use measured as (a) days of methamphetamine use and (b) methamphetamine-positive weekly saliva tests, during 12 weeks of active treatment relative to the placebo. The secondary hypotheses are that daily oral NAC delivered as a take-home medication will, relative to placebo:

- reduce the severity of methamphetamine dependence, the craving for methamphetamine, methamphetamine withdrawal symptoms and psychiatric symptoms (depressive symptoms, suicidality, psychotic symptoms and hostility)
- have an acceptable adverse event (AE) profile
- not significantly increase the use of other substances (including alcohol, tobacco, cannabis, heroin and cocaine).

## Methods/design

### Design

**Fig. 1:**
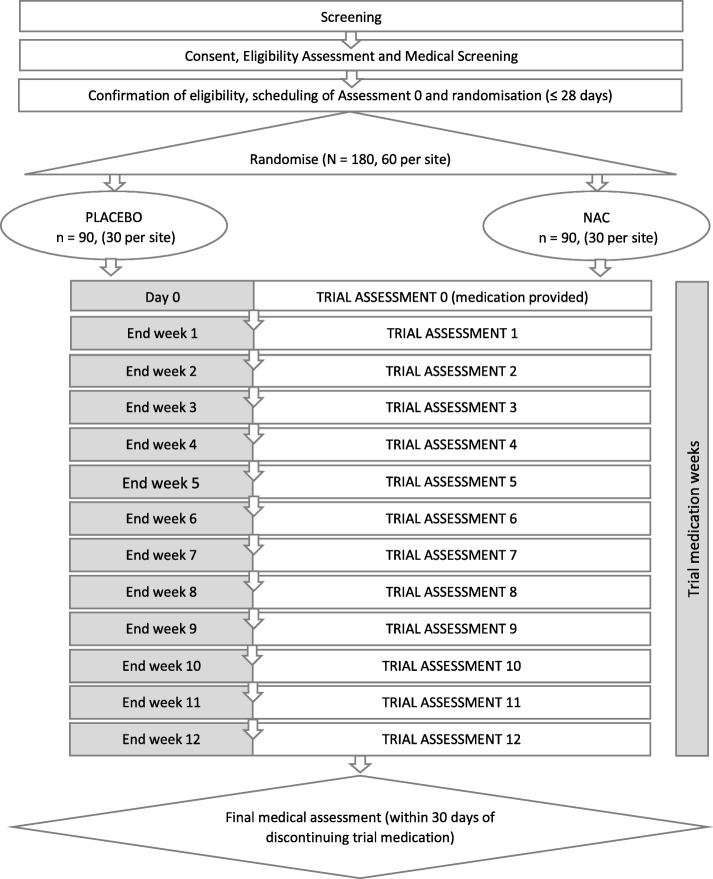
Trial flow diagram. NAC *N*-acetylcysteine

This is a three-site phase IIb randomised double-blindplacebo-controlledparallel-group trial. Participants will be recruited across three sites (*N* = 180; 60 per site) and randomly assigned (1:1) to receive either oral NAC 2400 mg daily or the placebo, for 12 weeks. Weekly assessments throughout the 12-week medication phase will be used to assess outcomes. The trial flow diagram is presented in Fig. 1. The trial protocol was developed in accordance with the Standard Protocol Items: Recommendations for Intervention Trials (SPIRIT) Statement (see Additional file 1 for the SPIRIT checklist).

### Randomisation and blinding

Participants are randomised to the treatment or placebo arms in a 1:1 ratio using a permuted block randomisation schedule with variable block sizes, stratified by site (Melbourne, Geelong or Wollongong), gender (male or female) and main route of methamphetamine administration in the month prior to recruitment (injecting or not injecting). The randomisation sequence was generated a priori using an in-house custom program written by WL. A random seed, assigned by the data and safety monitoring board (DSMB) statistician, was used to generate the final randomisation schedule using this program. Allocation to treatment condition (placebo vs. NAC) is based on the participant’s unique study identifier, this being assigned by trial staff sequentially within each stratum on randomisation.

All trial staff, the trial statistician (WL) and investigators are blind to treatment allocation. The randomisation schedule is held by the trial site pharmacy and the DSMB statistician. Allocation to trial arm is done by the trial pharmacist according to the randomisation schedule (provided by the DSMB statistician) using the unique study identifier provided by the trial staff. Packaging is identical to conceal treatment allocation. A removable adhesive label indicates whether the medication is placebo or NAC.

Unblinding is done at the discretion of the DSMB, the trial investigators or the treating medical physician in a medical emergency. Trial investigators can unblind individual participants via a password-protected online portal.

### Setting and study population

Participants are being recruited via outpatient settings in Melbourne (Turning Point and Eastern Health), Geelong (Barwon Health) and Wollongong (Illawarra Drug and Alcohol Service) in Australia. All participants are volunteers. They provide written informed consent prior to participation (see Additional file 2 for the participant information sheet and consent form). Inclusion criteria are that participants must be aged 18–60 years, meet DSM-IV criteria for methamphetamine dependence, be seeking to reduce their methamphetamine use but are not currently undergoing substance use treatment (including pharmacotherapy for substance use disorders), are not in need of acute care for psychiatric or other medical conditions, do not have a primary psychotic disorder, are able and willing to comply with the trial protocol, and have no contraindications for NAC. Contraindications for receiving the trial medication include: previous hypersensitivity to NAC; use of medications containing NAC or thought to be hazardous if taken with NAC (e.g. carbamazepine and nitroglycerin); a known or suspected active systemic medical disorder including cancer or other medical condition that may exacerbate the risk of AEs from NAC (recent gastrointestinal ulcers or renal stones, epilepsy or history of seizures, asthma or atopy); and, surgery within the past 28 days. Further, participants must not be pregnant (confirmed at the eligibility assessment), lactating or planning to fall pregnant during the trial.

### Intervention

Randomised participants will receive 2400 mg per day of oral NAC or a matching placebo (microcrystalline cellulose), taken as two 600 mg capsules every morning and evening for 12 weeks. The trial medication is supplied by Pharmaceutical Packaging Professionals (PPP) Pty Ltd. It is encapsulated in white capsules, size 00, and bottled in identical 200 cm^3^ white round plastic wide-mouth packer bottles (2.3 in. in diameter and 4.3 in. in height) fitted with eCAP™ lids (see Adherence). As a minimal intervention, all randomised participants are provided with a complementary self-help booklet, *On Ice* (National Drug and Alcohol Research Centre, University of New South Wales).

### Schedule of activities and study procedures

The trial flow diagram (Fig. 1) shows the schedule of activities.

#### Recruitment and screening

Participants are recruited through advertisements (e.g., Facebook, media activity, and flyers in needle and syringe programs) and word of mouth. They are screened by phone. Potentially eligible participants are consented by trial staff and then undergo a face-to-face eligibility assessment (1.5 to 2 h in total) to confirm their eligibility and to obtain information on demographics, drug use and psychiatric history. All participants undergo a medical assessment to confirm further their eligibility prior to randomisation. Randomisation must occur within 28 days of eligibility being confirmed. Eligible participants are provided with a list of local health service providers and are free to access drug treatment and other health services during the trial. Ineligible participants are offered referral to local alcohol and other drug services.

#### Assessments

The eligibility assessment and assessment 0 are conducted at the trial site clinic. Weekly assessments (assessments 1–12) are scheduled thereafter (with a leeway of −2 days to +4 days). These may be done at a public venue convenient to the participant (e.g., a café or shopping mall) to enhance retention. They take around 40 min each. Detailed contact information is collected on all participants and text message reminders are sent prior to appointments to enhance follow-up rates. Saliva samples are taken at each of the follow-up assessments using a Quantisal Oral Fluid Collection Device™. Participants undergo a final medical assessment within 30 days of completing the trial or on discontinuation of their medication. All participants are provided with referral information for alcohol and other drug treatment services at the close of the study.

#### Trial medication

Trial medication bottles are distributed to the trial site pharmacy in batches. On dispensing, the adhesive label indicating treatment allocation is removed by the pharmacist and placed in the participant’s pharmacy file. Trial medication is provided to the trial researcher who delivers the medication to the participant at assessment 0. Medication bottles are replaced at 3-week intervals. Participants are provided with feedback on their medication adherence at each assessment using eCAP™ technology (Med-ic eCAP™, Information Mediary Corp, see Adherence for details). The number of capsules remaining in each medication bottle is audited on return.

#### Safety monitoring

AEs are recorded by trial research staff at each assessment and reviewed by the trial physician. Face-to-face medical assessments are scheduled for all severe and serious adverse events (SAEs), and also at the discretion of the trial physician or at the request of the participant. Any ongoing AEs at the end of the trial are followed for at least 7 days, and ongoing SAEs for at least 30 days, with all AEs followed until stabilisation or resolution. All participants are entitled to compensation in accordance with the Medicines Australia Compensation Guidelines.

#### Discontinuation of medication and withdrawal from the trial

Participants are free to discontinue the trial medication or withdraw from the study at any time. Investigators may discontinue trial medication or withdraw a participant from the trial for the following reasons: pregnancy, no longer meeting the inclusion criteria, failing to take the medication for 7 or more days, significant non-compliance with the study protocol, disease progression that requires discontinuation of the trial medication or a clinical condition for which it would not be in the best interest of the participant to continue taking the trial medication. Follow-up assessments are completed regardless of medication discontinuation for all participants who remain consented into the study.

### Endpoints and assessments

Measures and measurement time points are presented in Table 1.

**Table 1:** Measures taken at each assessment

	Eligibility Assessment	Weekly trial assessments												
		0	1	2	3	4	5	6	7	8	9	10	11	12
Demographics, drug use history, CIDI (methamphetamine dependence), MINI (depression, psychotic disorders and mania), DIP (family history of psychotic disorders), RCQ, BCIS, BIS items, SAPAS	x													
Saliva samples			x	x	x	x	x	x	x	x	x	x	x	x
TLFB for days of methamphetamine use	x	x	x	x	x	x	x	x	x	x	x	x	x	x
Days of other substance use	x	x	x	x	x	x	x	x	x	x	x	x	x	x
Adverse events			x	x	x	x	x	x	x	x	x	x	x	x
Concomitant medications		x	x	x	x	x	x	x	x	x	x	x	x	x
Concomitant treatment		x	x	x	x	x	x	x	x	x	x	x	x	x
SDS		x	x	x	x	x	x	x	x	x	x	x	x	x
CEQ		x	x	x	x	x	x	x	x	x	x	x	x	x
AWQ		x	x	x	x	x	x	x	x	x	x	x	x	x
BPRS items		x	x	x	x	x	x	x	x	x	x	x	x	x
WPAI-GH		x	x	x	x	x	x	x	x	x	x	x	x	x
EQ-5D-5L		x	x	x	x	x	x	x	x	x	x	x	x	x
Health service use and criminal justice involvement		x	x	x	x	x	x	x	x	x	x	x	x	x
TSQM						x				x				x

*CIDI* Composite International Diagnostic Interview, *MINI* Mini International Neuropsychiatric Interview, *DIP* Diagnostic Interview for Psychosis, *RCQ* Readiness to Change Questionnaire, *BCIS* Beck Cognitive Insight Scale, *BIS* Birchwood Insight Scale, *SAPAS* Standardised Assessment of Personality – Abbreviated Scale, *TLFB* timeline followback, *SDS* Severity of Dependence Scale, *CEQ* Craving Experience Questionnaire, *AWQ* Amphetamine Withdrawal Questionnaire, *BPRS* Brief Psychiatric Rating Scale, *WPAI-GH* Work Productivity and Activity Impairment Questionnaire – General Health V2, *EQ-5D-5L* Five-dimension five-level version of EuroQol questionnaire, *TSQM* Treatment Satisfaction Questionnaire – Medication

#### Primary endpoint

The primary outcome is methamphetamine use. There are two measures of this endpoint:

1. Days of methamphetamine use during the 12-week active medication phase, assessed using the timeline followback method (TLFB) [31] and updated weekly. The outcome measure is the percentage of days on which methamphetamine is used during the 28 days prior to assessments 4, 8 and 12. The TLFB has 88% sensitivity, 96% specificity and 0.77 test–retest agreement for the use of amphetamines in the past 30 days [31].
2. The number of methamphetamine-positive saliva samples taken at assessments 1–12. Saliva is collected with an Alere Quantisal Oral Fluid Collection Device™ and the presence of methamphetamine confirmed using mass spectrometry with a detection cut-off of >25 mg/L.

#### Secondary endpoints

All secondary outcomes are assessed for the past week at assessments 0 to 12.

##### Methamphetamine craving

Methamphetamine craving is assessed using the total score on the Craving Experience Questionnaire (CEQ), which has been validated for various substances [32]. The original CEQ has ten items: three items corresponding to the urge to use the substance, three items corresponding to intrusive thoughts and four items corresponding to the sensory aspects of substance use (e.g., picture, taste, smell and feel). The sensory items were adapted for methamphetamine and combined into three items: (1) taste and smell combined, (2) picturing methamphetamine and (3) imagining what it would feel like to smoke or inject it. This gave a total of nine items, each with scores ranging from 0 to 10 with higher scores representing a more severe craving.

##### Severity of methamphetamine dependence

The severity of methamphetamine dependence is assessed using the total score on the Severity of Dependence Scale (SDS) [33]. The SDS is a five-item scale that yields scores from 0 (no dependence) to 15 (most severe dependence). It has good internal consistency for amphetamine use (Cronbach’s alpha from 0.81 to 0.89) [33] and has been validated against a diagnosis of severe amphetamine dependence using the Composite International Diagnostic Interview (CIDI) [34].

##### Methamphetamine withdrawal symptoms

Methamphetamine withdrawal symptoms are assessed using the total score on the Amphetamine Withdrawal Questionnaire (AWQ) [35]. The AWQ is a ten-item questionnaire that rates various symptoms associated with amphetamine withdrawal on a five-point Likert scale. Scores range from 0 to 40 with higher scores indicating higher severity. The AWQ has a test–retest reliability of 0.79 and has been validated against other measures of withdrawal [35].

##### Psychiatric symptoms

Psychiatric symptoms are assessed using the Brief Psychiatric Rating Scale (BPRS) [36]. Symptom domains are rated against anchor points from 1 (nil) to 7 (extremely severe). Scores of 4 or greater indicate symptoms of clinical significance or pathological intensity. The BPRS has an inter-rater reliability of 0.83 when quality assurance procedures are followed [37]. The following endpoint definitions were used:

- Psychotic symptoms: A score of 4 or greater on any of the BPRS items of suspiciousness, unusual thought content and hallucinations
- Hostility: A score of 6 or 7 on the BPRS hostility item
- Depression: A score of 4 or greater on the BPRS depression item
- Suicidality: A score of 4 or more on the BPRS suicidality item

### Other assessments

#### Polysubstance use

The polysubstance use measure is the percentage of days using drugs from other classes (tobacco, alcohol, cannabis, cocaine, ecstasy, hallucinogens, inhalants and heroin) for the 28 days prior to assessments 0, 4, 8 and 12, assessed weekly using the TLFB, and averaged across drug types.

#### Adverse events

AEs will be assessed at each weekly assessment using open-ended questions and may include any other events that come to the attention of the trial staff during the trial. The outcome will be the number and percentage of participants reporting AEs and SAEs by System Organ Classification, coded according to the Medical Dictionary for Regulatory Activities. AEs will be counted once only for a given participant. The event counted will be the event with the highest severity (coded mild, moderate or severe).

#### Adherence

eCAP™ (Med-ic eCAP™, Information Mediary Corp) will be used as an objective measure of medication adherence. eCAP™ lids fitted to the medication bottles used record the times when a bottle is opened, which are downloaded at each assessment using a near-fieldcommunication-enabled smartphone and on bottle return using a CertiScan RFID desktop Reader (Med-ic Certiscan Version 2.5.1; Information Mediary Corp 2014–2018). Adherence will be the percentage of compliant doses, based on bottle opening, with dosing timeframes being morning (until 12 noon) and night (until midnight), or equivalent 12-h intervals.

#### Treatment satisfaction

The Treatment Satisfaction Questionnaire for Medication (TSQM) [38] will be completed at assessments 4, 8 and 12.

#### Concomitant treatments

All medications, including supplements, taken during the trial and all treatments related to either substance use or other mental health conditions received during the trial will be recorded on a template adapted from the National Institutes of Health’s concomitant medications form [39].

#### Health Economics

Data for costing are being collected to facilitate a subsequent economic evaluation of the intervention. These costing data include the EuroQol (EQ-5D-5L) [40] version 2.1, the Work Productivity and Activity Impairment Questionnaire – General Health V2 (WPAI-GH), and health service use and contact with the criminal justice system, which are assessed using a structured questionnaire. The data are updated weekly throughout the 12-week trial medication period.

#### Demographics, drug use and psychiatric history

Demographics, drug use and psychiatric history data are collected at the eligibility assessment. Demographics and drug use questions are based on the Opiate Treatment Index [41] and previous drug treatment trials [42], and include days of use for all major drug types in the past month. Current DSM-IV methamphetamine dependence is confirmed using CIDI [43]. The Mini International Neuropsychiatric Interview (MINI) [44] for Schizophrenia and Psychotic Disorders Studies, English Version 7.0.1, is used to screen for psychotic and affective disorders. Other measures include family history of psychotic disorders, assessed using the Diagnostic Interview for Psychosis (DIP) [45], the Readiness to Change Questionnaire (RCQ) [46], the Beck Cognitive Insight Scale (BCIS) [47], four items from the Birchwood Insight Scale (BIS) [48], and the Standardised Assessment of Personality – Abbreviated Scale (SAPAS) [49].

### Governance and ethical considerations

#### Quality control

All trial researchers will receive training in the trial assessment procedures. BPRS ratings are reviewed weekly, and a selection of ratings (10%) are audio-recorded and rated by an independent trained BPRS rater to establish inter-rater reliability. Primary endpoints are double entered. Weekly researcher meetings and monthly investigator meetings are held to monitor recruitment, follow-up, data collection and other trial procedures. A delegate from the coordinating sponsor institution (Curtin University) is responsible for conducting site visits (prior to initiation, during the trial and at trial completion) to audit compliance with the protocol and the accuracy and completeness of the data.

#### Data safety and monitoring board

The DSMB meet at least semi-annually and include individuals with expertise in the clinical topic area (methamphetamine and other substance use disorders), randomised clinical trials and biostatistics. The current DSMB also includes a consumer/community representative. The role of the DSMB is to review participant safety, recruitment, accrual, retention and withdrawal. The DSMB can recommend trial suspension, modification or discontinuation.

#### Data management

Personal information obtained from participants is stored securely and separately from trial data at the trial site and is accessible only to trial staff from that site. Participants’ trial data are de-identified using a unique study identifier. Saliva samples are de-identified and destroyed after analysis. De-identified trial data are entered onto a centralised secure (password protected) online electronic database, Research Electronic Data Capture (REDCap), which is hosted on a secure Curtin University server. De-identified trial data are available to all trial investigators. All analyses are based on de-identified data and will be published in a way that participants cannot be individually identified. Trial participants will be provided with a summary of their results at the close of the study. Otherwise, trial data are confidential and will be released in identified form only with the permission of the participant or as required by law. Trial data will be stored securely for 15 years after study completion; destruction of study material thereafter will be in accordance with local ethics approvals.

#### Ownership and publication

The ownership and permissions relating to the trial data are managed under a multi-institutional contractual agreement between the study sponsors, which is available on request from RM. The decision to publish information from the study will be made jointly by the trial investigators. Authorship will be in accordance with the guidelines of the International Committee of Medical Journal Editors. There are no other restrictions on the publication of trial results.

#### Governance and ethics

The N-ICE trial (Universal Trial Number U1111–1210-1224) has been registered prospectively with the Australian and New Zealand Clinical Trials Registry (ACTRN12618000366257) and has been approved by all relevant human research ethics committees: Eastern Health (E21–2017), Barwon Health (17/202), the University of Wollongong and Illawarra Shoalhaven Local Health District Health (2017/549) and Curtin University (HRE2018–0205). The trial is funded by the National Health and Medical Research Council (NHMRC) under project grant 1128147 and is run under a co-sponsor arrangement between the participating institutions and the trial sites. The participating institutions are Curtin University, Deakin University, the University of Wollongong, Monash University, the Burnet Institute, La Trobe University and the University of Newcastle. Curtin University is the lead coordinating institution (National Drug Research Institute, Curtin University, GPO Box 1987, Perth WA 6845; phone +61 89,266 1602, facsimile +61 89,266 1611). The trial sites are in Melbourne (Turning Point and Eastern Health), Geelong (Barwon Health) and Wollongong (Illawarra Drug and Alcohol Service). Oversight is provided by an independent DSMB, which operates under a charter agreed by the DSMB members (available on request from RM). Curtin University coordinates the overall conduct of the trial, including communication with the DSMB, and monitors the study at all sites to ensure compliance with the protocol. Each site is individually responsible for ensuring compliance with good clinical practice and site-specific ethics protocols, including communicating any changes of the trial protocol to local governance authorities and participants.

### Statistical considerations

#### Sample size estimation

The sample size of 90 participants per treatment arm is designed to detect a mean between-group difference of 3.5 days versus 6.0 days of methamphetamine use per month during the 12-week trial medication period with at least 80% power at *p* < 0.025 (0.05 / 2 to accommodate two primary outcome measures) and with 20% attrition (i.e., a final sample of at least 72 participants per treatment arm). The expected effect size is based on data from the Methamphetamine Treatment Evaluation Study (MATES) [42]. Participants who did not receive treatment in that cohort showed a reduction in methamphetamine use from a mean (standard deviation) of 12.8 (7.4) days per month at baseline to 6.0 (6.7) days per month at the 12-weekfollow-up. Our estimate of a minimal clinically meaningful reduction in use is based on outcomes for outpatient counselling derived from MATES: 11.2 (8.8) days per month to 3.5 (6.9) days per month at 12 weeks. Our estimate of 20% attrition is based on our previous research [42, 50, 51]. No interim analyses are planned. Statistical power was estimated using the SAMPSI command in Stata (Version 14.2, StataCorp LLC, College Station, Texas).

#### Efficacy analysis

All tests will be two-sided with significance set at *p* < .025 for each of the two primary endpoints and *p* < .01 for each of the secondary endpoints. Significance for all other tests will be set at *p* < .05. The analyses will be conducted blind to condition.

##### Analysis of primary outcomes

Intention-to-treat analyses will be conducted for the primary outcomes. The effect of the trial medication on days of methamphetamine use (percentage of days used in the past 28 days) will be tested using a baseline (assessment 0) vs. medication phase (three repeats: assessments 4, 8 and 12) by treatment condition (NAC vs. placebo). Unobserved days (e.g., due to hospitalisation or incarceration) will be censored. The effect of NAC on methamphetamine-positive saliva will be tested using a main effect for treatment condition (NAC vs. placebo) on saliva test results (positive vs. negative) from assessments 1–12 (12 repeats).

##### Analysis of secondary outcomes

Analysis of secondary outcomes will be based on a modified intention-to-treat dataset that includes only participants who completed assessment 0 and at least one follow-up assessment (i.e., assessments 1–12). The effect of NAC on each secondary outcome will be tested using a baseline (assessment 0) vs. medication phase (assessments 1–12) by treatment condition (NAC vs. placebo) interaction.

All effects will be tested with mixed effects models with repeated measures for each measurement time point. Heterogeneity in variance and treatment effect between sites will be tested for by seeing whether the model fit is significantly improved by including a random intercept for site and a random coefficient for the main effect by site, respectively. If no significant heterogeneity is found, the more parsimonious model will be retained. Missing data will be imputed using multiple imputation by chained equations implemented in Stata in the analysis of the primary outcomes only. Final details of the analysis, including methods of imputation and covariates, will be outlined in a statistical analysis plan prior to unblinding of the data. An additional statistical plan for a health economic evaluation will be developed prior to undertaking any such analysis.

## Discussion

The N-ICE trial is the first randomised controlled trial to assess the efficacy of NAC as a pharmacotherapy for methamphetamine dependence. It will test whether NAC can produce a clinically meaningful reduction in methamphetamine use. It will assess whether there are any beneficial effects of NAC on related clinical endpoints, including psychiatric symptoms, and it will document adherence to NAC and the safety profile of NAC as a take-home medication in this population. Thus, the trial will improve our understanding of the potential utility of NAC in clinical practice to reduce methamphetamine use.

One clear potential benefit of NAC, if it is found to be safe and effective, is that it can be administered as a take-home medication, making it a potentially scalable and cost-effective treatment option. A significant impediment to the benefits of NAC, or any other take-home medication, would be poor compliance with dosing regimens. Thus, we are using electronic monitoring (eCAP™) to record the time and date of each occasion a bottle is opened to provide an objective measure of adherence. Electronic monitoring through devices such as eCAP™ is a relatively recent technological development [52] that has not yet been widely adopted in medication trials for illicit substance use. This technology will provide important data on the viability of prescribing take-home medications in illicit substance-using populations and it will also allow us to assess the implications of sub-optimal dosing on treatment effects and safety in this population.

Novel aspects of our trial procedures are the direct community engagement strategy to recruit participants and the outreach methods for follow-up assessments within the community, in so far as these are practical and safe. We have taken this approach because most people who are dependent on methamphetamine do not engage with specialist drug treatment services [53], and this has made recruitment to and retention in stimulant pharmacotherapy trials difficult [54]. Rather than relying on recruiting patients from existing clinical services and requiring that trial participants come to fixed sites for treatment, we use well-honed fieldwork methods that have been successfully applied in survey and cohort research involving people who use methamphetamine [42, 50, 55]. We hope that the implementation of these outreach methods within the clinical trial framework will increase engagement with consumers and improve retention.

A challenge that we faced in developing this trial protocol was the lack of standardised and readily available evidence-based treatment options for methamphetamine dependence. This precluded a treatment comparison arm or the provision of treatment as usual to trial participants. Instead, we provide all participants with a form of minimal intervention (an information booklet) and referral information for health services, which they are free to access throughout the trial period. To counter the risk of bias from concomitant treatment, we record all concomitant medications and psychosocial treatments for substance use and other mental health problems. We will compare treatment arms on these exposures and, where differences exist, we will adjust for these in the main analysis.

In summary, this trial is important because it tests a novel, affordable, available and tolerable non-agonist pharmacotherapy option for methamphetamine dependence, which, if effective, has the potential to be a much-needed, cost-effective and scalable treatment option for this population. NAC has the potential to be prescribed by primary health-care providers as a take-home medication and used as an adjunct to existing drug treatments. The lack of patent restrictions and its current availability as a prescription medication and dietary supplement will facilitate its uptake. Given the lack of approved medications currently available for managing methamphetamine dependence [3–5] and the challenges involved in translating effective psychological interventions into practice settings [56], the discovery of a safe and effective pharmacotherapy would fill an important gap in treatment options for methamphetamine use.

### Trial status

The current version of the protocol that has been approved by the relevant human research ethics committees is 3.0 (29 May 2018). The first participant was randomised on 9 July 2018. By 9 May 2019, 77 participants had been randomised. It is anticipated that recruitment (*N* = 180) will be completed by the end of 2019.

## Additional files

Additional file 1: SPIRIT Checklist. (DOC 121 kb)

Additional file 2: Participant information sheet and consent form. (DOCX 66 kb)

## Data Availability

The datasets generated during the current study are not publicly available due to privacy laws but are available from the corresponding author on reasonable request and pending appropriate ethics approval and the approval of the investigator team.

## Abbreviations

AE: Adverse event
AWQ: Amphetamine Withdrawal Questionnaire
BCIS: Beck Cognitive Insight Scale
BIS: Birchwood Insight Scale
BPRS: Brief Psychiatric Rating Scale
CEQ: Craving Experience Questionnaire
CIDI: Composite International Diagnostic Interview
DIP: Diagnostic Interview for Psychosis
DSMB: Data and safety monitoring board
EQ-5D-5 L: Five-dimension five-level version of EuroQol questionnaire
MATES: Methamphetamine Treatment Evaluation Study
MINI: Mini International Neuropsychiatric Interview
NAC: *N*-acetylcysteine
NHMRC: National Health and Medical Research Council
RCQ: Readiness to Change Questionnaire
SAE: Serious adverse event
SAPAS: Standardised Assessment of Personality – Abbreviated Scale
SDS: Severity of Dependence Scale
TLFB: Timeline followback
TSQM: Treatment Satisfaction Questionnaire for Medication
WPAI-GH: Work Productivity and Activity Impairment Questionnaire – General Health
