# Supplementary material for: A study protocol for the N-ICE trial: A randomised double-blind placebo-controlled study of the safety and efficacy of N-acetyl-cysteine (NAC) as a pharmacotherapy for methamphetamine (“ice”) dependence
Source: Trials. 2019 Jun 4;20:325. doi: 10.1186/s13063-019-3450-0 (PMC6549263; doi:10.1186/s13063-019-3450-0)
Supplement: Supplementary file 2 — Participant information sheet and consent form. (DOCX 66 kb) [file 13063_2019_3450_MOESM2_ESM.docx]

Site:

***[Insert site name and logo]***

******

***Facsimile: 03 5221 3429***

Participant ID:

Participant Initial: ________

**Participant Information Sheet and Consent Form**

| **Title** | The N-ICE trial: A randomised controlled trial of the safety and efficacy of N-Acetyl-Cysteine (NAC) as a pharmacotherapy for methamphetamine (“ice”) dependence |
| --- | --- |
| **Short Title** | The N-ICE Trial |
| **Protocol Number** |  |
| **Project Sponsor** | Curtin University |
| **Collaborating Institutions** | Deakin University, Burnet Institute, Monash University, The University of Wollongong |
| **Principal Investigator** | Associate Professor Rebecca McKetin |
| **Associate Investigators** | Dr Olivia Dean, Prof Daniel Lubman, Prof Paul Dietze, Dr Peter Higgs, Dr Peter Kelly, Dr Alyna Turner, Dr Brendan Quinn, Prof Gregory Carter, Prof Amanda baker, Dr Barbara Sinclair, Dr David Reid, Prof Michael Berk, Dr Ruth Collins, Dr Wenbin Liang |
| **Location** | *[Insert site name]* |

**Introduction**

You are invited to take part in this research study of the drug N-Acetyl-Cysteine (NAC) as a possible new treatment for methamphetamine (“ice”) dependence. To be able to participate you

1. want to reduce your methamphetamine use,
2. are not currently in drug treatment, and
3. are not currently taking any medication for your methamphetamine use.

This Participant Information Sheet and Consent Form tells you about the research project. It explains the tests and treatments involved. Knowing what is involved will help you decide if you want to take part in the research. Please read this information carefully and ask questions about anything that you don’t understand or want to know more about. Before deciding whether or not to take part, you might want to talk about it with a relative, friend or your local doctor.

Before you agree to participate in this research study, it is important that you read and understand the following explanation of the study. It describes the purpose, procedures, benefits, risks, discomforts, and precautions associated with the study. It describes your rights as a participant, including the right to withdraw from the study at any time. It is important to understand that no guarantees or assurances can be made regarding the results of the study. It is also important to know that during the trial **you will receive either the NAC or the placebo (dummy pills) twice a day for 12 weeks.** You will have an equal chance (1 in 2) of receiving either of the two treatments.

If you decide you want to take part in the research project, you will be asked to sign the Consent Form. By signing the Consent Form, you indicate that you understand the information in this sheet and that you give your consent to take part in the research project. You will be given a copy of this Participant Information and Consent Form to keep.

**What is the purpose of this study?**

This study aims to examine whether N-Acetyl-Cysteine (NAC) can help people who want to reduce and manage their methamphetamine use. It is believed that NAC has effects that lead to improvements in brain systems which may help to reduce the craving for methamphetamine and aid in the management of mental health. NAC has have previously been trialled for methamphetamine dependence and found to reduce craving for methamphetamine and be well-tolerated. However, we do not know whether NAC can reduce methamphetamine use or improve mental health in people who use methamphetamine. Importantly, we cannot guarantee that you will receive any benefit from taking NAC.

NAC is currently an approved drug in Australia and other parts of the world to treat conditions such as Panadol overdose and lung and respiratory diseases such as bronchitis, asthma, and cystic fibrosis. However, this study is using NAC is an experimental treatment. This means that it is not yet an approved treatment for methamphetamine dependence in Australia or other parts of the world.

This research project aims to recruit approximately 180 participants at sites in NSW and Victoria. The project has been organised by a collaboration of researchers from Hospitals, Drug and Alcohol Clinical Services, and Universities throughout Australia, and is funded by the National Health and Medical Research Council.

**What does participation in this study involve?**

If you agree to participate, we will ask you to go through a baseline interview and medical assessment to make sure you are eligible to take part in the 12 week trial. During the face-to-face baseline interview, which will take about an hour to complete, the trial researcher will ask you questions about your demographics, drug use, treatment history, psychological health and emotional well-being. We will also collect your contact information so that we can keep in touch with you throughout the trial. The trial researcher will then refer you to the trial doctor for a medical assessment to make sure that the study is a good fit for you (for example: to confirm that you don’t have any illnesses, and (if female) that you’re not pregnant using a urine/pregnancy test). The whole assessment will take about 2 hours.

We will contact you in the week after this initial assessment to let you know whether you are eligible and to make sure you are still happy to participate in the trial. If you are eligible, you will be randomised into one of the two study groups: either the NAC or the Placebo group. The NAC group will take 2 capsules in the morning and 2 in the evening, each capsule containing 600mg NAC. If you are in the placebo group you will take an identical set of capsules that will contain inactive substances. Every three weeks the trial researcher will give you a new bottle of the study medication to take home. It is very important that you take this study medication as instructed and bring your medication bottles back to the clinic, even if you have missed some doses. You will not know whether you are receiving NAC or placebo. The study staff will also not know whether you are receiving NAC or placebo. This information is kept with the hospital who are providing your medication, and can be accessed in an emergency by providing the information on your study participation card to your doctor.

During the 12 week trial there will be weekly face-to-face meetings with the trial researcher either here at the trial clinic, or if you prefer, at a safe and public location that is convenient to you, such as a local café. These meetings will take about one hour. At each meeting you will be asked a series of questions about your drug use and your physical and mental health. You will also be asked to provide a sample of your saliva to be tested for methamphetamine. After the end of the 12 weeks, there will be a final medical assessment with the trial doctor to see if you’re feeling well and to discuss any side effects you may have experienced during the trial. This will take about 30 minutes and needs to occur within 1 month of finishing the trial.

At some of the weekly assessments, we would like to audio record a short section of the interview. This is for training purposes and to check that our assessments are accurate. This is voluntary. If you do not wish to have your interview recorded please let your trial researcher know.

**Will participating in the study cost me anything?**

Participation in this study won’t cost you anything, nor will you be paid. All medication, tests and medical care required as part of the study will be provided to you free of charge. However, you will be reimbursed up to $30 for every visit to cover out-of-pocket expenses in participating in the research.

**What are the possible benefits of taking part?**

From this research, we hope to learn more about whether NAC can help people reduce their methamphetamine use and improve their mental health. This information may eventually be used to help improve treatment options for people who use methamphetamine. However, we cannot guarantee that you will receive any benefits from participating in the research.

**What are the possible risks and disadvantages of taking part?**

N-Acetyl-Cysteine (NAC) is generally safe and very few people experience side effects that would make them want to stop taking it as a medication. The components in NAC may have very mild potential side effects, such as gastrointestinal upset (diarrhea, constipation, nausea and heartburn), but serious side effects are very rare. In addition to gastrointestinal upset, NAC has been rarely reported to cause aggravation of asthma, stomatitis (inflammation of the mouth), rhinorrhea (runny nose), fever, and sedation. As with any medication, allergic reaction is also a possibility. However, this tends to be uncommon and when allergy or other side effects do occur they are usually very mild. As this is an experimental use of the study drug, there may be additional unknown or unforeseen risks.

During the study you will be closely monitored by our trial researchers and doctors. **Tell your trial researcher and/or your study doctor immediately about any new or unusual symptoms that you get or if you have any concerns regarding the medication.** Your study doctor will discuss the best way of managing any side effects with you. You will be withdrawn from the study if you develop any severe or serious side effects that your study doctor thinks are related to NAC.

**Pregnancy risks**

The effects of NAC on the unborn child and on the newborn baby are not known. Because of this it is important that you are not pregnant or breast feeding and do not become pregnant during the course of the study. You must not participate in the study if you are pregnant or trying to become pregnant or breast feeding. If you are male you should not father a child while taking the trial medication.

Both male and female participants are advised to use effective contraception during the course of the study. You should discuss methods of effective contraception with your doctor. If you do become pregnant whilst participating in the study you should advise your treating doctor and researcher immediately. Your treating doctor will withdraw you from the study and advise on further medical attention should this be necessary. You must not continue to take the study medication if you become pregnant.

**Other risks**

This research project involves the collection of information about your drug use. Your participation in the study, and any relevant related medical information, will be kept on your medical record, and it may be accessible to other medical staff (e.g. if you presented to hospital your doctor at the hospital may have access to this information). Participation in the research project also includes saliva analysis to determine the presence of methamphetamine. The test may reveal evidence that you have used illegal drugs. We will keep your information confidential and will generally not disclose this information without your consent, but there may be circumstances where we are required by law to do so. In that case, the information could be potentially used against you in legal proceedings or otherwise. To our knowledge, researchers at this institution have not been required by law to provide information. If we were ever required to do so, we would do our best to notify you before disclosing it.

**What if new information becomes available?**

All new findings discovered during the course of this research that may reasonably influence your willingness to continue participation in this study will be made known to you as soon as they become available. This new information may mean that you can no longer participate in this research. If this occurs, the person(s) supervising the research will stop your participation. In all cases, you will be offered all available care to suit your needs and medical condition.

**Can I have other treatments during this study?**

During the trial you will be free to participate in other treatments for your drug use and mental health, however, you must tell us if you do so. Sometimes your participation in other treatments may mean that you are unable to continue in the trial. For example, your treatment may mean that you are unable to complete the study procedures (e.g., if you were in a residential treatment you may not be allowed to take NAC, or you may not be able to do the assessments) or it may not be safe to keep taking NAC if you are prescribed another medication (e.g., some medications can increase the risk of side-effects from NAC). Your trial doctor will make this decision.

You must tell the trial researcher and study doctor about any medical treatments that you will have to receive or undergo during the study period (such as elective surgery), medication you may be taking, including non-prescription medications, vitamins or herbal remedies and any changes to these during your participation in the study. It is very important to let them know, because there is a possibility that other medicines or drugs may interact with NAC and cause you harm.

**What are the alternatives to participation?**

You do not have to take part in this research project to receive treatment at this health service. Other options are available, which your study doctor will discuss with you before you decide whether or not to take part in this research project. You can also discuss the options with your local doctor.

**What do I do if I wish to withdraw from the study?**

Your participation in this study is entirely voluntary. You do not have to take part in it. If you do take part, you can withdraw at any time without having to give a reason. Whatever your decision, please be assured that it will not affect your relationship with this service [*insert site name]* or any of the doctors here.

If you wish to withdraw your consent, please notify your trial researcher immediately. We ask that if you do withdraw from the study that you attend a final medical assessment to provide a final assessment of your health and complete a withdrawal form. If you are having any health effects from the trial medication we will keep in touch with you for up to one month after you stop taking the trial medication. After this, the study doctor and relevant study staff will not collect any additional study related information from you. If you indicate that you would like your study data destroyed, all of your trial data (such as baseline and follow-up assessment data) will be deleted from the electronic study database and any paper files will be destroyed. You should be aware though that a record of your participation in the study, your study identification, personal details, medical assessment details and eligibility will be retained and these may be available on your medical record.

**Could this study be stopped unexpectedly?**

Your trial doctor may end your participation in this research program for any reason that they may feel is appropriate. These may include but are not limited to unacceptable side effects, injury, if you get pregnant (if applicable) or are trying to get pregnant, a medical condition or medication which may place you at risk of further complications if you continue to participate, failure to take the medication as instructed, or termination of the study by the investigators or for other administrative reasons.

**What happens when the study ends?**

Should you want treatment for methamphetamine dependence after the study is finished, you will be offered a referral to the clinic’s usual treatment services. The study drug NAC is currently not available for treating methamphetamine use except for in clinical trials. When the study is finished, you should speak to your doctor about the treatment options available to you.

If you would like to find out about the results of the research, please advise your study researcher who will be able to post or email you a one-page summary after the analysis is completed (expected to be towards the end of 2019).

You can also agree to be contacted after the end of this study about any follow-up studies, or other related studies, that you may be interested in participating in. This is completely voluntary and it has no bearing on whether or not you can participate in this study.

**What will happen to information about me?**

By signing the consent form you consent to the study doctor and relevant research staff collecting and using personal information about you for this study. Any information obtained in connection with this study that can identify you will remain confidential. All information that is collected about you for the purposes of this study will be marked with a code instead of your name. This includes any audio-recordings that we make during the interview. A list with your name and matching code will be kept at the clinic, with only non-identifiable information being sent off site. Your personal information will only be used for the purpose of this research project and will not be disclosed unless you have given us permission or if disclosure is required by law. Circumstances where we may be required to disclose your confidential data include if we receive a subpoena to do so (i.e., we are ordered by a court to present evidence), in the event that we are required to report about child abuse or neglect, and if we are concerned about your immediate safety or the safety of others around you (e.g., if you are suicidal or express intent to harm someone).

Information about you may be obtained from your health records held at this and other health services for the purpose of this research. By signing the consent form you agree to the study team accessing health records if they are relevant to your participation in this research project. Information about your participation in this research project may be recorded in your health records.

It is anticipated that the results of this research project will be published and/or presented in a variety of forums. The results of the research project may also include additional analysis of the data for related research purposes (e.g., to see if the medication is cost-effective, to examine health and well-being amongst people who use methamphetamine). In any publication and/or presentation, information will be provided in such a way that you cannot be identified.

After the study has been completed, all study-related documents will be stored securely for 15 years in line with national research guidelines, and you may access that information if required. After the 15 year period your paper records will be shredded and destroyed and the electronic files deleted.

**What will happen to my saliva samples?**

You will be required to provide weekly saliva samples during the study. Your saliva samples will be de-identified (labelled with a code instead of your name) and sent to a central laboratory. The samples will be tested for the presence of methamphetamine and will be destroyed 3 months after the results are known.

**Pregnancy test**

If you are a female you will be required to do a pregnancy test using a urine sample at the start of the study. The results of this test will be documented and the pregnancy test will be destroyed.

**What if something goes wrong?**

If you suffer any injuries or complications as a result of this research project, you should contact the study team as soon as possible and you will be assisted with arranging appropriate medical treatment. If you are eligible for Medicare, you can receive any medical treatment required to treat the injury or complication, free of charge, as a public patient in any Australian public hospital.

**Complaints and compensation**

You may have a right to take legal action to obtain compensation for any injuries or complications resulting from the study. Compensation may be available if your injury or complication is caused by the drugs or procedures, or by the negligence of any of the parties involved in the study. In the event of loss or injury, the parties involved in this research project have agreed to conditions of compensation in accordance with the Medicines Australia Compensation Guidelines which is available online at the Medicines Australia website:

<https://medicinesaustralia.com.au/policy/clinical-trials/indemity-and-compensation-guidelines/>.

**Who has reviewed the study?**

All research in Australia involving humans is reviewed by an independent group of people, called a Human Research Ethics Committee (HREC). This clinical trial has been reviewed and given approval by [insert].

**How do I get more information?**

The outline of the research study has been described to you in this consent form. If you would like more information about the study or if there is any matter about it that concerns you, either now or in the future, do not hesitate to ask one of the members of the clinical trial team or your doctor. Before deciding whether or not to take part you may wish to discuss the matter with a relative or friend or with your doctor. You should feel free to do this.

If you have any questions about the study at any time, feel free to contact the researchers:

Associate Professor Rebecca McKetin:

***[insert site coordinator details]***

If you seek emergency care, or if hospitalisation is required, please alert your treating physician that you are enrolled in a research study being conducted by Associate Professor Rebecca McKetin**.**

If you have any complaints about any aspect of the project, the way it is being conducted or any questions about rights as a research participant, then you may contact:

***[Insert Ethics committee contact details]***

***[Insert site name and logo]***

**Participant Consent Form**

**Study Title** The N-ICE trial: A randomised controlled trial of the safety and efficacy of N-Acetyl-Cysteine (NAC) as a pharmacotherapy for methamphetamine (“ice”) dependence

- I have read the Information Sheet and I understand the purpose of the clinical trial, what is involved, what data is being collected, any possible risks, inconveniences or discomforts involved, and what will be done with the data upon completion of the clinical trial.
- I have been given the time and opportunity to ask questions about the clinical trial and any questions I have asked have been answered clearly and to my satisfaction. I have also been given the opportunity to discuss this clinical trial with a person not connected to the clinical trial.
- I give consent for samples of my saliva to be taken weekly during the trial for the purpose of this clinical trial as outlined in the Participant Information form.
- I understand that all information provided by me is treated as strictly confidential and will only be shared with the clinical trial team and not be released by the clinical trial team unless required to do so by law.
- I know that I may withdraw from the trial at any time without having to give any reason or affecting my current or future medical treatment.
- I understand I will receive a copy of the participant information and signed consent form to keep.
- I understand and consent to those regulatory authorities and other organisations referred to in the participant information having access to my confidential information.
- I agree to participate in this research and give my consent voluntarily.

**In addition:**

- I give my consent for the trial researchers to contact me for up to two years after the end of this trial about any follow-up studies or other additional studies that I may be interested in taking part in. I understand that this is completely voluntary and that I have no obligation to participate in any other studies even if I agree to be contacted.

Yes No

______________________________________________ ________________

Printed Name of Participant Initial

______________________________________________ ________________

Signature of Participant Date (participant to date)

**Declaration by Researcher**

In addition to allowing the participant to read and discuss this Participant Information Sheet and Consent Form with myself and anyone the participant feels is relevant, I have given a verbal explanation of the research project, its procedures and risks and I believe that the person participant has understood that explanation.

Name of Researcher (please print) _________________________________________________

Signature_____________________________________Date___________________________

***[Insert name of institution and logo]***

Form for Withdrawal of Participation

**Study Title:** The N-ICE trial: A randomised controlled trial of the safety and efficacy of N-Acetyl-Cysteine (NAC) as a pharmacotherapy for methamphetamine (“ice”) dependence

I hereby wish to WITHDRAW my consent to participate in the clinical trial described above and understand that such withdrawal WILL NOT disadvantage me in any way.

Check preferred option:

I would like all of my study assessment data to be destroyed

I am happy for the researchers to use my study assessment data collected so far

______________________________________________ ________________

Printed Name of Participant Initial

______________________________________________ ________________

Signature of Participant Date (participant to date)

______________________________________________ ________________

Printed Name of Investigator Initial

______________________________________________ ________________

Signature of Investigator Date (Investigator to date)
